# Constraints on the Genetic and Antigenic Variability of Measles Virus

**DOI:** 10.3390/v8040109

**Published:** 2016-04-21

**Authors:** Shannon M. Beaty, Benhur Lee

**Affiliations:** Icahn School of Medicine at Mount Sinai, New York, NY 10029, USA; shannon.beaty@icahn.mssm.edu

**Keywords:** measles, paramyxovirinae, antigenic drift, genotype, genomic stability, genetic variation, mutation rate, amino acid substitution, viral envelope proteins

## Abstract

Antigenic drift and genetic variation are significantly constrained in measles virus (MeV). Genetic stability of MeV is exceptionally high, both in the lab and in the field, and few regions of the genome allow for rapid genetic change. The regions of the genome that are more tolerant of mutations (*i.e.*, the untranslated regions and certain domains within the N, C, V, P, and M proteins) indicate genetic plasticity or structural flexibility in the encoded proteins. Our analysis reveals that strong constraints in the envelope proteins (F and H) allow for a single serotype despite known antigenic differences among its 24 genotypes. This review describes some of the many variables that limit the evolutionary rate of MeV. The high genomic stability of MeV appears to be a shared property of the *Paramyxovirinae*, suggesting a common mechanism that biologically restricts the rate of mutation.

## 1. Introduction

Measles virus (MeV) is an enveloped virus with a nonsegmented, negative-sense RNA genome that is 15,894 nucleotides in length. It belongs to the genus *Morbillivirus* in the *Paramyxovirinae* sub-family. The organization of the MeV genome is similar to that of most members of the *Paramyxovirinae*: the genome contains six transcription units, which are separated by nontranscribed trinucleotide intergenic sequences and are flanked by 3′ leader and 5′ trailer sequences at the genome ends (Figure 2A). The MeV genome encodes a total of eight proteins. The six structural proteins are the nucleocapsid protein (N), phosphoprotein (P), matrix protein (M), fusion protein (F), attachment protein (H), and the large error-prone RNA-dependent RNA polymerase protein (L). Two additional nonstructural proteins (C and V) are encoded in the P transcription unit. While the C protein is translated from an overlapping reading frame within the P gene [1], the V protein is initiated from the same start codon as P, but a frameshift is created by mRNA editing. The outcome is that P and V share an N-terminal domain of 231 amino acids, but differ in their C-terminal domains (276 and 69 amino acids, respectively) [2].

## 2. Genotypic Heterogeneity in Measles Virus

Wildtype MeV isolates are genetically heterogeneous, with 24 genotypes recognized to date (A, B1–B3, C1–C2, D1–D11, E, F, G1–G3 and H1–H2) [3,4]. Only eight of these genotypes have been detected in recent years (B3, D3, D4, D6, D8, D9, G3, and H1), while five genotypes (B1, D1, E, F, and G1) are considered inactive since they have not been detected for more than 25 years [5,6]. The World Health Organization has established that measles genotyping requires a minimum sequence from the highly variable carboxy-terminal 450 nucleotides of N—a region of the gene termed N-450—or the entire protein-coding region of the H gene [3]. Some antigenic differences have been identified between viruses belonging to different genotypes [7,8] and specific genotypes have been causally linked with certain outbreaks or endemic transmission [4], but the clinical significance of the molecular and genetic differences between genotypes is not known [9,10].

An association has not yet been established between specific genotypes and differences in severity of disease, such as progression to measles inclusion body encephalitis (MIBE) and subacute sclerosing panencephalitis (SSPE), which are rare, fatal neurological conditions that result from persistent measles infection in the brain [11]. The genotype that is detected in persistently infected brain tissues is usually consistent with the genotype that was circulating at the time when the patient experienced acute measles infection. However, to our knowledge, viruses belonging to only 10 different genotypes have been isolated from SSPE patients (A, B, C1, C2, D1, D3, D4, D5, D7, E and F), while only three genotypes have been found in MIBE patients (A, B3 and E) [12,13,14,15,16,17,18,19]. That the latter occurs exclusively in immunocompromised subjects may explain that a vaccine strain (genotype A) [13] has been found to be the cause of at least one case of MIBE, whereas vaccine strains of measles have not been isolated in any cases of SSPE [20]. In summary, given the rarity of neurological complications of MeV infection (estimated to be between 1 in 1700 and 1 in 3300 cases of measles for children below five years of age [21]) and the lack of an appropriate animal model, a causal genotype-phenotype relationship might be impossible to establish even if the heterogeneity in the severity and clinical outcome of MeV infection might be influenced by the genotype of the virus at some level.

## 3. Serology, Genotypes, and Antigenic Diversity

Despite its genotypic diversity, measles has only one serotype, which implies a high degree of similarity in the surface antigens across all MeV strains. *In vitro* seroneutralization experiments have shown that a broad range of measles genotypes, likely all, can be neutralized by serum samples from vaccinees or individuals who have experienced a natural measles infection, although neutralizing titers can vary for different genotypes [7,22]. The single serotype nature of MeV is further evidenced by the fact that vaccination confers protective immunity against all known genotypes, even though all vaccine strains are members of a single genotype (genotype A). Furthermore, despite the presence of differing endemic genotypes around the world, effective mass vaccination campaigns have dramatically reduced the number of measles-related deaths globally and interrupted endemic transmission in large geographic areas [10,23]. In countries with high vaccination coverage and high seroprevalence rates, the overwhelming majority of measles cases occur in individuals that are unvaccinated or incompletely vaccinated [24]. The geographic variations in incidence are likely the result of varying degrees of vaccination coverage, and not the degree of protection that vaccination confers against various endemic genotypes.

In contrast with neutralization studies performed using serum from vaccinated individuals, vaccine-derived monoclonal antibodies have indeed revealed differences in their ability to neutralize different genotypes. A number of monoclonal antibody studies have sought to provide a structural and biochemical basis for the antigenic differences between genotypes, and perhaps more importantly, to elucidate the antigenic similarities that allow for a single measles serotype in spite of genotypic variation [25,26,27,28,29,30,31]. While vaccination elicits both F-specific and H-specific neutralizing antibodies, those directed against the H protein have a much larger contribution to virus neutralization [32], and as such have been the primary focus of antigenic studies. A recent study by Tahara *et al.* (2013) found that five distinct epitopes on the H protein are conserved across a virus panel spanning eight genotypes, and monoclonal antibodies that bind two of these epitopes, were highly effective for neutralizing viruses of all genotypes tested. One of these effective neutralizing epitopes is located in an epitope in the head domain involved in binding to the signaling lymphocytic activation molecule (SLAM) and antibody binding to this epitope was shown to inhibit SLAM binding. The other conserved neutralizing epitope is not involved in SLAM binding and antibody binding at this site is proposed to interfere with H-F interaction. Furthermore, an additional epitope was identified and characterized to be an effective neutralizing epitope in several, but not all, of the genotypes tested [33,34]. The existence of highly conserved epitopes that are targets for neutralizing antibodies is suggestive of structural and/or functional constraints on the measles H protein, which would prevent the emergence of escape mutations and contribute to the single serotype nature of MeV. A recent study by Lech *et al.* (2013) found that escape mutant viruses generated against a variety of neutralizing anti-H monoclonal antibodies were all neutralized effectively by polyclonal serum, indicating that MeV may need to carry escape mutations in multiple neutralizing epitopes of H for it to escape neutralization by polyclonal serum [35]. *In vitro* mutagenesis experiments support that the H protein is structurally constrained [36] and the long-lived immunity conferred by vaccination or natural infection suggests that MeV does not undergo any significant antigenic drift. In fact, there are surprisingly few regions across the entire MeV genome that allow for rapid change. These regions are discussed in the latter sections of this review.

## 4. High Genetic Stability of MeV in the Lab and in the Field

The genetic stability of measles is exceptionally high, and it has been observed that it undergoes remarkably little sequence variation over long periods of time, both in laboratory settings and in the field. In several studies on MeV genetic stability, comparison of genomic sequences obtained after passaging *in vitro* revealed either complete sequence identity with the seed stock [37,38] or a single nucleotide change between two working stocks that had a widely divergent passage history [39]. Even the most variable sequence in the MeV genome, N-450, appears to be very stable over the course of *in vitro* passaging, irrespective of the cell type used for growth [39]. As reviewed by Aktories, *et al.* (2009), a high level of genetic stability has also been observed in field isolates of MeV and there is very little variation in the N and H gene sequences of viruses isolated from the same chain of transmission [40]. Moreover, sequencing of viral isolates from the same genotype that were collected several years apart has revealed very little genetic change [41,42]. Rima *et al.* (1997) estimated the rate of mutation for MeV in the field to be 5 × 10^−4^ substitutions per base per year [41] and a similar estimate of 4 × 10^−4^ was made by Jenkins *et al.* (2002) [43] (Table 1). The substitution rate of MeV is significantly lower than estimates for many other RNA viruses such as human immunodeficiency virus type 1 (HIV-1), influenza virus A, foot-and-mouth disease, human enterovirus 71, which have substitution rates in excess of 1.6 × 10^−3^ substitutions per base per year [43,44,45,46,47].

Despite the multiple mutations that accumulate in strains of MeV that are associated with more severe neurological outcomes, these strains are no more genetically unstable than acute or vaccine strains of the virus. In fact, while high stability is seen in all strains of MeV, persistent viruses from SSPE and MIBE patients are perhaps even more genetically stable. Substitutions in SSPE associated viruses can be extensive, but taking into consideration the substantial latent period of ~7–10 years, the rate of molecular evolution for SSPE strains is estimated to be 3.4 × 10^−4^ substitutions per base per year [19]. This is similar to estimates for acute strains, while other estimates have calculated the substitution rate of SSPE strains to be even lower than in circulating viruses [9]; however, since these estimates do not take into account differences in the replication rates of acute and persistent viruses, it is difficult to directly compare their genetic stability. Nucleotide substitutions in SSPE-associated virus accumulate in particular regions of the genome, namely in the matrix and fusion genes, while the character of mutations are indicative of biased hypermutation [76]. Thus the difference between acute and persistent strains cannot be attributed to a higher overall rate of genetic change *per se*, but rather might be influenced by qualitative differences in the type of mutations that arise or variation in as yet unidentified host factors. The mechanistic underpinnings of viral persistence are currently an area of active investigation.

**Figure 1:**
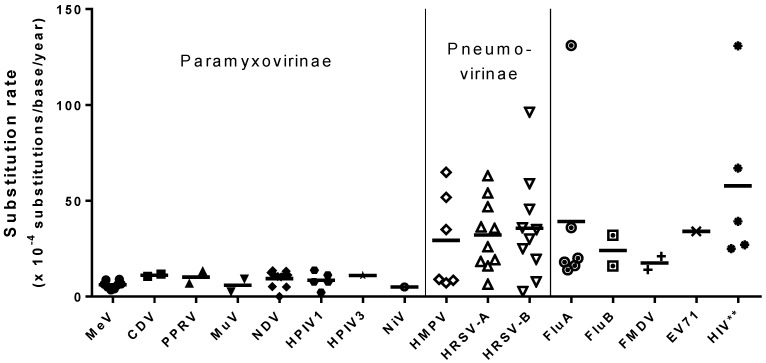
Reported substitution rates for selected viruses with RNA genomes. Each point represents a reported estimate of the substitution rate for the indicated virus. Estimates include measurements made from one or more gene sequences (N, F or H/HN/G genes) and/or the entire genome sequence. Horizontal bars indicate the mean for each virus. ** Human immunodeficiency virus (HIV) is a retrovirus and is included in this figure for comparison.

Interestingly, high genetic stability has been observed for many other paramyxoviruses in addition to MeV, and reported substitution rates are similar across the *Paramyxovirinae* subfamily [50] (Figure 1). This high genetic stability appears to be a property of the *Paramyxovirinae* subfamily only, and does not extend to the *Pneumovirinae*. A much higher substitution rate has been observed for both subgroups of human respiratory syncytial virus (HRSV) as well as human meptapneumovirus (HMPV), although the F and N genes of HMPV are notably more genetically stable than the attachment protein (G) [62,63,64,75]. Many paramyxoviruses have an extraordinarily low level of variation among strains. Rima *et al.* (2014) compared the sequences from 15 different strains of parainfluenza virus type 5 (PIV5), and found remarkably low diversity among them, in spite of the isolates being derived from a broad range of host species (humans, monkeys, pigs, and dogs) over a time period spanning several decades [77]. Similarly, mumps virus (MuV) also has very little diversity among strains of the same genotype [78]. Cui *et al.* (2013) compared the SH and HN gene sequences from 39 MuV strains belonging to seven different genotypes. In both genes, sequences from the same genotype were identical, regardless of being collected sporadically over several years from patients with or without neurological symptoms and without any epidemiological links between the cases [79]. Minimal genetic distance has been observed between different isolates of both Nipah (NiV) and Hendra (HeV) viruses, independent of host species or year isolated [80]. During the 1998 outbreak of NiV, sequences of isolates obtained from humans, pigs, and bats of the *P. hypomelanus* genus were found to be nearly identical, with only 56 nucleotide differences across the >18kb viral genome [81,82]. A bat isolate of NiV that was obtained six years later from a *P. vampyrus* bat was found to have only minor sequence variation from all known Malaysian isolates [61]. The exceptionally high genomic stability shared by most *Paramyxovirinae* suggests a common mechanism that biologically constrains the rate of mutation.

## 5. Mechanisms of Mutagenesis and Constraints on Mutation

MeV and other paramyxoviruses exhibit much higher genetic stability than most RNA viruses and there are numerous factors that could be contributing to this difference. For many RNA viruses, extensive studies have been done to determine mutation rates due to intrinsic factors, rather than selection. For RNA viruses, the intrinsically error-prone polymerase and lack of proofreading mechanisms result in a much higher rate of nucleotide misincorporation than that observed for DNA viruses, and *in vitro* measurements of the error rate for polymerases from RNA viruses ranges from 1 × 10^−6^ to 1 × 10^−3^ substitutions/site/replication [83,84,85]. The *in vitro* mutation rate of the MeV polymerase is similar to those of other RNA viruses, with estimates ranging from 1.8 × 10^−6^ to 9 × 10^−5^ substitutions/site/replication [86,87]. Thus, there is no evidence to suggest that there is a dramatic difference in the intrinsic error rate of the MeV RNA polymerase. Instead, the high error rates of the MeV polymerase likely produce large numbers of mutants during replication, but relatively few mutations are tolerated. This process of purifying selection, in which genetic diversity is narrowed through the elimination of genomes carrying deleterious mutations, is stronger in RNA viruses than DNA viruses [88]. However, the various selection pressures that drive this phenomenon in MeV are not well understood.

The reasons for such shallow genetic diversity in MeV and other *Paramyxovirinae* are not clear, and constraints potentially operate at many levels. Structural and biological pressures that limit change in specific regions of the genome are discussed in latter sections of this review. All viruses in the *Paramyovirinae*, but not the *Pneumovirinae*, are governed by the “rule of six”, in which their genomes must be an exact multiple of six nucleotides in length to replicate efficiently [89]. The rule of six restricts genomic tolerance for insertions or deletions, and these kind of mutations are rarely observed in MeV [90]. The genome is further constrained by hexameric phasing, which describes the periodic manner in which the N protein interacts with the genome. For each group of six nucleotides bound by the same N molecule, the position of a given nucleotide within that group corresponds to phases 1 to 6. The phase of some transcriptional elements is highly conserved in paramyxoviruses, which might allow these sequence elements to be better accessed by the viral polymerase complex [89,91]. For example, the phase of the transcriptional start site for the various mRNAs is remarkably conserved across every genus of the subfamily [89]. If phase is an important feature for viral transcription, it might function as a constraint on synonymous mutations that disrupt the conserved phasing pattern.

Recombination, which is an important mechanism for rapid genetic change in many viruses, might also be inhibited in part by the rule of six. Although recombination is common among positive-stranded RNA viruses that encode their own RNA polymerase [92], it rarely occurs in negative sense RNA viruses (NSVs). Infrequent recombination in NSVs might be attributed to the fact that neither genomic nor anti-genomic RNA is ever naked, to low rates of co-infection, or to selection against the low fitness of recombinants—although it is not clear why NSV recombinants should be less fit than recombinants in other lineages [93]. There is some evidence that recombination in MeV is possible [94], but studies on the recombination rate of viruses isolated from natural infections have failed to reveal any MeV recombinants [93,95]. Other factors that might constrain substitution rates are those that affect recognition by innate immune responses, such as avoiding the formation of double-stranded RNA structures with complementary cellular RNAs, including micro RNAs (miRNAs) [77]. Additionally, biases against specific dinucleotides [41] and codon usage [96,97] exert an effect on translational efficiency, and could provide a significant constraint on the occurrence of synonymous mutations.

RNA editing by adenosine deamination, resulting in biased hypermutation, may play a role in the generation of the sequence variation in the MeV genome. Members of an enzyme family known as adenosine deaminases that act on RNA (ADARs) catalyze conversion of adenosine to inosine in the negative-strand RNA genome, which then pairs preferentially with cytosine residues during template copying [98]. It has not been directly determined if ADARs are responsible for the sequence transitions observed in MeV substitution events, but their involvement in generating sequence diversity is supported by indirect evidence [98]. Analysis of variation in the MeV genome has revealed a pattern indicative of ADAR-mediated hypermutation, including a preponderance of U-to-C substitutions, mutations that appear in clusters, and a high ratio of nucleotide transitions to transversions [99,100]. It has been shown that ADAR1 acts as a proviral host factor for MeV infection, as viral growth in ADAR1-deficient cells is reduced while virus-induced apoptosis and interferon-β (IFN-β) induction is enhanced. However, the precise role of ADAR enzymes in the MeV lifecycle remain largely unknown [101,102]. The prevalence of hypermutation has been found in sequence comparisons between different strains of MuV, PIV5 and HRSV [77,103,104], suggesting that this mechanism of genetic change might be a common feature among all *Paramyxoviridae*.

Interestingly, it has been reported that the degree of hypermutation may vary between acute and persistent virus strains, particularly in the reading frame of the M protein [9,105]. Extensive U-to-C substitutions have been observed in sequences of M genes derived from patients with SSPE and MIBE [85,100,105,106,107,108]. In the M sequence of viral RNA isolated from the brain of an MIBE patient, ~50% of the U residues were changed to C [106]. Wong *et al.* (1991) found that 80% of the nucleotide differences between the M gene sequences of a persistent virus and a closely related acute strain involved U-to-C transitions, and biased hypermutations were determined to be responsible for all but one of the numerous missense genetic changes predicted to cause amino acid substitutions [108]. Differences in the type of nucleotide substitutions that arise in the evolution of acute and persistent virus could indicate distinct selection pressures or mutational mechanisms involved in the development of persistence. While the clustered distribution of mutations suggests an active mechanism of mutation, rather than mere accumulation of polymerase errors [9], the biological implications and exact mechanism behind biased hypermutation in MeV is not currently known. *Henipaviruses* (another genus of paramyxoviruses) can also establish persistent infections that result in central nervous system (CNS)-confined relapsing encephalitis that can occur up to 11 years after recovery from the primary infection [109,110]. No sequences from relapsing henipavirus encephalitis cases have been deposited in the database, but analysis of such sequences would help determine if the biased hypermutation seen in SSPE is a general property of persistent paramyxovirus infection in the brain or unique to the biology of MeV.

## 6. Variation in the Mutational Tolerance across Coding Regions

While the factors that constrain mutations and genetic variation in MeV are not well defined, the uneven distribution of mutational tolerance across the genome may provide some insight into the selective forces that drive its overall genomic stability. High tolerance for mutations in some regions of the genome is evidenced by the genetic variability observed in field isolates or passaged laboratory strains and a corresponding high tolerance for substitutional or insertional mutagenesis. *In vitro* transposon mutagenesis of the MeV genome by Fulton *et al.* (2015) found that viral mutants containing an insert in genomic locations that are known to be highly variable were frequently recovered and grew to higher titers than mutants with an insert in highly conserved regions [36] (Figure 1A,B). Taken together along with biochemical evidence, these findings highlight several regions of the genome that are inherently plastic and indicate structurally flexible domains of the encoded proteins. These regions are discussed below: 

### 6.1. The N Protein

The C-terminal domain of the N protein, known as NTAIL, is one of the regions found to be most tolerant of insertional mutagenesis [36] and, with the exception of two conserved hydrophobic patches, NTAIL is extremely variable across MeV isolates [111]. Indeed, as previously discussed, genotyping of viral isolates is based partially on sequences from this region of the genome due to its extremely variable sequence. The structural flexibility implied by high mutational tolerance of NTAIL is supported by protein sequence analysis and biochemical evidence. NTAIL is an intrinsically disordered domain [112,113], and the disordered nature of the C-terminal domain of N appears to be a conserved feature within members of the *Paramyxovirinae* subfamily [114,115]. Biochemical evidence indicates that unbound NTAIL exists as a dynamic population of interconverting conformers and it undergoes α-helical folding upon binding to the P protein [111]. While the C-terminal domain of the N protein is hypervariable in wildtype strains, the N gene is one of the most conserved genes among MeV vaccine strains, displaying a much higher level of conservation than the P/V/C, M, and H genes. For wildtype isolates as well as vaccine strains, the N terminal domain of the N protein is much more conserved than NTAIL, and sequence comparison of all MeV vaccine strains found only four cumulative nucleotide changes in the region coding for the N-terminal 400 amino acids [116].

### 6.2. The P/V/C Proteins

**Figure 2:**
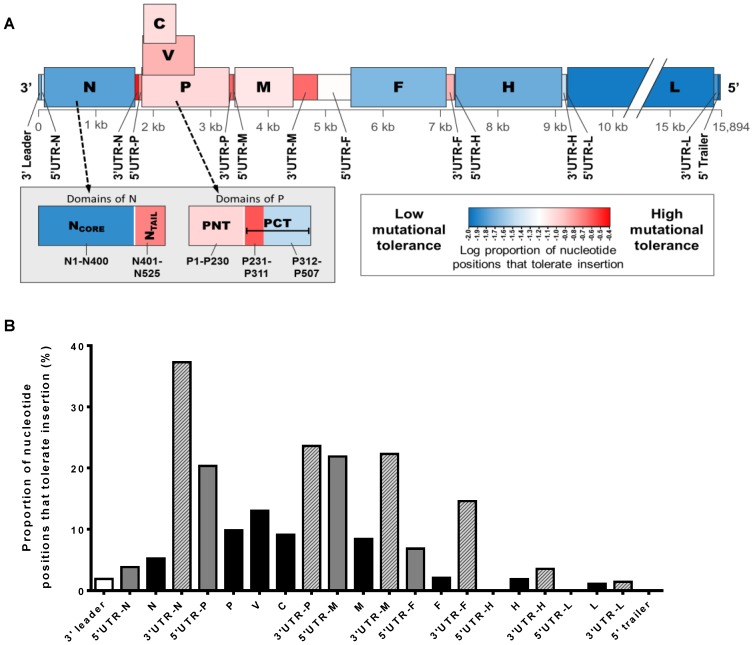
Tolerance for insertional mutagenesis in different regions of the MeV genome. The number of positions that tolerate transposon-mediated insertional mutagenesis in each region of the genome were normalized to the length of the region to determine insertional tolerance. (**A**) Schematic representation of the MeV genome organization, with each region colored according to its mutational tolerance. UTR = untranslated region. Grey inset box shows the mutational tolerance of different domains within N and P, with residue numbers indicated below each respective domain; PNT = N-terminal domain of P; PCT = C-terminal domain of P; (**B**) Quantification of tolerance for insertional mutation in different genomic regions. Data obtained from Fulton *et al.* (2015) [36].

High sequence variability is also frequently observed in the P gene [117,118]. Nucleotide substitutions are often observed in the P gene after passages of MeV in tissue culture, and in wildtype viruses, the P genes demonstrate a higher level of variation than the corresponding N genes [119,120,121]. The shared N-terminal domain of V and P was found to be more variable than the carboxyl termini in wildtype MeV isolates and vaccine strains [116,117], which indicates a higher degree of conformational flexibility in the common N-terminal domains of the P and V proteins. This is further supported by the finding that a green fluorescent protein (GFP) tag can be added to the N-terminus, but not the C-terminus, of the P and V proteins without significantly compromising function [122]. Furthermore, the poorly conserved N-terminal domain of P (PNT, amino acid (aa) 1–230) was found to be highly tolerant of transposon-mediated insertional mutagenesis [36] (Figure 2). On the other hand, the C-terminal domain of P (PCT, aa 231–507) is highly tolerant of insertions in the first 80 residues (aa 231–311), while it is relatively intolerant of insertions within the C-terminal 197 residues (aa 312–507). The latter includes the coiled-coiled domain essential for P oligomerization (aa 344–411), as well as the XD domain (aa 459–507) important for inducing the correct folding of the disordered NTAIL mentioned above [112,123,124]. The C-termini of both P and V are well conserved and relatively resistant to insertional mutagenesis, which is likely because this region regulates P-L, P-N, and P-P protein interactions as well as contain critical domains required for viral transcription and replication (P) or control of the innate immune response (V), respectively [116,125]. For several *Paramyxovirinae* genera, including Morbilliviruses, Henipaviruses, and Respiroviruses, a third protein (C) is encoded within the P gene from an overlapping reading frame. Like P and V, the C protein is also intrinsically disordered, highly variable among different strains, and substitutions in the C gene have a high frequency of coding changes [116,126].

### 6.3. The M Protein

The sequence diversity observed in sequences of the M gene from wildtype viruses is relatively low [127]. M gene sequences are well conserved across different strains for other paramyxoviruses as well, including NDV and MuV [128,129]. Considering the sequence conservation found in the M gene of wildtype strains, it is surprising that M is not required for viral replication and mutants that do not express the M protein are viable *in vitro*, albeit with lower replicative fitness [107,130]. Furthermore, the M gene was found to have a high tolerance for mutagenic insertions [36]. In vaccine strains, the M gene has a higher substitution rate and a higher proportion of coding changes than the N, F, or L genes [116]. In addition, viruses isolated from SSPE patients usually have a highly mutated M gene, with mutations that often create premature stop codons that completely eliminate M protein expression [9]. In contrast, mumps viruses isolated from cerebrospinal fluid of acute viral meningitis cases revealed almost no mutations in the M gene [129], indicating that the accumulation of mutations in M is not a generalizable property of paramyxovirus infections of the brain *per se*. Rather, M mutations may reflect the selective pressures involved in the establishment of persistent infections in the brain as discussed above (Section 5). Altogether, it appears that there is some fitness advantage to conservation of the MeV-M gene sequence, but the protein itself has a certain degree of structural plasticity and can change rapidly in response to different selection pressures.

### 6.4. The F and H Proteins

Variation might be expected in the viral fusion (F) and attachment (H) envelope glycoproteins due to immune selective pressures; however, both MeV envelope proteins are extremely stable. F is more conserved than the M or H genes, and the proportion of coding changes is lower. There are few differences between the F proteins of different wildtype or vaccine strains. Identical amino acid sequences of the F protein have been found between the attenuated Zagreb strain, the wildtype Edmonston strain, and the more recent wildtype IC-B isolate, despite a great disparity in the year they were isolated and a widely divergent passage history [116,121,131], which demonstrates a high level of conservation in F. Although the F protein is very highly conserved among wildtype and vaccine strains, particularly in important functional domains and motifs, such as the cytoplasmic tail, cleavage site, and fusion peptide [116], viruses isolated from SSPE patients usually display considerable mutations in the fusion F gene, usually restricted to the cytoplasmic tail domain [9]. For many viruses, including influenza A virus, sequence diversity readily develops in surface-exposed protein epitopes, facilitating evasion of the adaptive immune response. Although escape mutations that allow for evasion of seroneutralization can arise in both F and H proteins, antigenic evolution does not occur at an appreciable rate in either of the MeV envelope glycoproteins [51,132]. The intolerance for insertional mutation observed in the F and H genes suggests that this might be the result of structural constraints that prevent the development of antigenic diversity [36].

The level of variation observed in the H protein is extremely low [51,133]. As discussed above, measles H is quite antigenically stable over long periods of time, and this lack of antigenic drift makes the H gene sequence suitable for genotyping. As a whole, the H gene was found to be extremely resistant to insertional mutations, which is attributed to structural constrains on the protein itself [36]. Three proteinaceous cellular receptors have been identified for MeV—SLAM (or CD150), CD46, and Nectin-4 (or PVRL4)—and MeV-H has overlapping but distinct receptor binding sites for each of these, located on the side of its six-bladed beta-propeller head domain [134,135,136,137,138,139,140,141]. Amino acid residues that are involved in receptor binding are highly conserved among wildtype and vaccine strains that have common receptor usage [134,142,143,144,145]. All amino acid residues that are involved in SLAM- and Nectin-4 binding are conserved in Edmonston wildtype and all vaccine strains [116] and high conservation of these residues extends across the *Morbillivirus* genus [143]. Additionally, the cytoplasmic tails of the H proteins (aa 1−34) are completely conserved among all vaccine strains [116]. Comparison of vaccine strains with a wildtype strain showed that although the number of substitutions in the H gene is low, the ratio of coding changes is very high (92%) [116], which indicates that these mutations may be driven by selection pressure and could reflect attenuation or tissue culture adaptation. The use of a proteinaceous cellular receptor(s) for viral entry and the attendant larger receptor-envelope interaction interface might be thought to impose a greater constraint on the mutational tolerance of MeV-H compared with the attachment protein of other paramyxoviruses that use sialic acid as a receptor. However, a similar level of antigenic stability has been observed for the attachment proteins (HN) of sialic acid-using paramyxoviruses, such as mumps [78] and PIV5 [77,146]. Perhaps the complex molecular choreography that leads from receptor binding to fusion-protein triggering [147,148] might be the dominant constraint on the genetic stability of MeV-H (and paramyxovirus attachment proteins in general) regardless of whether the receptor is protein or glycan based. Future comparisons as to how tolerant HN-bearing paramyxoviruses are to insertional mutations in their HN protein will be highly informative.

### 6.5. The L Protein

The L open reading frame (ORF) stands out as the most conserved gene and it is highly refractory to insertional mutations [36,149]. The L gene is considerably more conserved among vaccine strains than any of the other genes, with only 75 cumulative changes across the 6549 nucleotide-long gene, the majority of which do not change the coding sequence [116,150]. Sequence conservation of the L gene appears to be a common feature of many other paramyxoviruses, including NDV, PIV5, and MuV, where a lower rate of substitution and a predominance of synonymous mutations has been observed [77,78,151]. In addition to the binding domains that allow L to interact with other MeV proteins, six discrete functional domains have been characterized for the L proteins of MeV and their sequences are well conserved across paramyxovirus L proteins [152,153]. The functions of these domains include all enzymatic activities required for transcription and replication. The functional domains in L are arranged within a larger protein structure that is comprised of at least two defined structural domains, which can be expressed as split protein fragments containing dimerization tags without compromising normal protein function [149]. Of the 16 positions in L that vary among the vaccine strains, the majority are located between the conserved domains [116]. The insertion of an epitope tag can be tolerated at two interdomain positions in L (residues 615 and 1708), but none of the highly conserved functional domains have been found to tolerate insertions [36,149]. The lack of transposon insertions in the interdomain regions [36] known to be tolerant to epitope tags [149] may reflect the fitness costs associated with such insertions. In summary, the extremely low tolerance for mutation in any of the functional domains of the L protein likely reflects some degree of structural inflexibility and underscores the interdependence of the various structural domains in mediating the critical role of L in viral transcription and replication.

## 7. Variation in Mutational Tolerance across Non-Coding Regions

Non-coding RNA comprises nearly 11% of the 16-kb MeV genome and exhibits some degree of conservation across wildtype and vaccine strains [116,154,155,156,157]. Many specialized sequence motifs known as cis-acting regulatory sequences have been identified within the non-coding regions of the MeV genome, and these elements have known or proposed functions in genome replication, genome packaging, translation, mRNA synthesis, processing, and editing [155]. Known *cis*-acting regulatory elements are extremely well conserved across all MeV strains [116,154,155,156]. The six transcription units are flanked by 3′ leader and 5′ trailer sequences located at the genomic termini. Comparison of viral sequences derived from various vaccine strains and wildtype viruses belonging to many different genotypes has revealed an extremely high level of conservation in the leader and trailer [116,155,157]. This conservation is likely driven in large part by the critical role of the genomic termini in viral replication; while both the leader and trailer contain critical regulatory elements such as encapsidation signals and promoters for genomic and antigenomic transcription, respectively, the 3′ leader also contains a promoter sequence for the initiation of mRNA synthesis [155]. Thus, considerable deviation from the consensus leader/trailer sequences could impair proper promoter recognition by the polymerase complex and severely attenuate replication. Indeed, for HPIV3, a related paramyxovirus in the *Respirovirus* genus, mutations in the leader sequences have been implicated in contributing to an attenuated phenotype [158].

In the intergenic sequences, the 3′ and 5′ untranslated regions (UTRs) that flank each transcription unit contain highly conserved gene-end (GE) and gene-start (GS) signals that mediate transcription termination and reinitiation during mRNA synthesis [154,155]. The UTRs have been shown to play an important role in the regulation of transcription and translation of viral genes in paramyxoviruses [159,160,161], and mutations within these regions could alter protein expression by affecting transcript initiation, translation efficiency or mRNA stability. Alteration of non-coding regions in MeV and canine distemper virus (CDV), a closely related morbillivirus, has been shown to modulate virulence through transcriptional control of gene expression [162,163]. In MeV, the intergenic region between the M and F genes is unusually long (~1 kb) compared with that of other paramyxoviruses (generally <0.5 bp), but data regarding the functional elements of the UTRs contained in this region are difficult to reconcile into a unifying hypotheses. The 3′UTR of M and the 5′UTR of F are thought to play important roles in viral replication and pathogenicity. In CDV, the M/F intergenic region was shown to potently modulate virulence through transcriptional regulation of F protein expression [162]. Furthermore, expression of the F protein is reduced in vaccine strains of MeV, which is thought to be due to changes in the 5′UTR of F and may reflect a mechanism of attenuation [164]. However, portions of the 5′UTR of MeV-F are dispensable for viral replication in cell culture [165], and while substitutions in the 5′UTR of CDV-F dramatically affected pathogenesis, deletions in this region had no effect on viral replication or the course of disease *in vivo* [162].

Interestingly, despite the critical role of the UTRs in viral replication and pathogenesis, they are surprisingly tolerant of insertional mutations. Insertional mutagenesis found the non-coding regions to be the most highly mutable regions in the genome, particularly the N/P, P/M, and M/F intergenic regions [36] (Figure 1). Furthermore, the 3′UTR of each gene is generally more tolerant of insertions than the corresponding 5′UTR. The biological significance of this difference awaits future investigations. Furthermore, the M/F intergenic region can be quite variable and has been found to contain the most substitutions in a comparison of non-coding regions from wildtype and vaccine strains [155,166], which is consistent with the finding that the M/F intergenic region is most accepting of transposon insertions [36]. In addition, the M/F intergenic region is often the most variable region of the genome viral sequences derived from persistent MIBE and SSPE strains and persistent isolates sometimes contain insertions or deletions in addition to substitutions [14,167]. This observation suggests that mutation of the non-coding regions could be mechanistically involved in the development of persistence, but this remains to be determined experimentally.

## 8. Conclusions

Genetic variation and antigenic drift are significantly constrained in MeV, and there are undoubtedly many variables that contribute to its limited evolutionary rate. This high genetic stability appears to be a shared property of the *Paramyxovirinae*, suggesting a common biological restriction on the rate of mutation that has not yet been defined. Genetic epistasis and the complex interactions between different viral proteins are likely responsible for some of the variation in mutational rates across different regions of genome. Structural, functional, and genetic constraints are particularly high in the envelope and polymerase proteins of MeV, the former of which probably contributes to its single serotype nature and the long-lived immunity conferred by vaccination or natural infection. In general, the mutational tolerance for insertions across the MeV genome is consistent with what is known about the genetic variability of MeV and the structure-function of each ORF. A revealing finding from the whole genome interrogation studies using insertional mutagenesis is that the 3′UTR of each gene is generally more tolerant of insertions than the corresponding 5′UTR, with the differences being most marked for the N, F, H, and L genes. This suggests a critical role for 5′UTRs—perhaps a regulatory function—that is ripe for future studies. Importantly, differences in the degree of tolerance to insertional mutagenesis revealed in the Fulton *et al.* (2015) study reflect the relative fitness costs compared with insertions in other nucleotide positions, as mutants were allowed to replicate in competition with each other during cell culture passages. Thus, a given genomic position might tolerate an insertion when examined in isolation, but may not be enriched relative to more fit mutants in this kind of whole-genome interrogation study. Future investigations are needed in order to address the more complex questions surrounding qualitative differences in mutations that arise under distinct growth conditions and how these differences relate to attenuation, virulence, or the development of viral persistence.

## Figures and Tables

**Table 1:** **Reported substitution rates for RNA viruses.** The range of substitution rates encompasses reported measurements made from any gene or entire genome sequence for the indicated virus. ** Human immunodeficiency virus (HIV) is a retrovirus and is included in this table for comparison.

Family or Subfamily	*Genus*/Species	Substitution Rate Substitutions/Base/Year (× 10^−4^)	References
**Paramyxovirinae**	*Morbillivirus*		
	MeV	3.4–9.02	[19,43,48,49,50,51]
	CDV	10.53–11.65	[50,52]
	PPRV	6.9–13.4	[53]
	*Rubulavirus*		
	MuV	2.5–9.168	[43,50]
	*Avulavirus*		
	NDV	5.04–13.5	[54,55,56]
	*Respirovirus*		
	HPIV-1	2.2–13.7	[43,57,58,59]
	HPIV-3	11.0	[60]
	*Henipavirus*		
	NiV	5	[61]
**Pneumovirinae**	*Metapneumovirus*		
	HMPV	7.12–64.9	[62,63,64]
	*Pneumovirus*		
	HRSV-A	6.47–63.0	[43,65,66,67,68,69,70,71,72]
	HRSV-B	2.7–96.2	[43,65,67,68,70,71,72,73]
**Orthomyxoviridae**	IFVA	13.9–131.0	[43,44,46,74]
	IFVB	16–32.0	[43,75]
**Picornaviridae**	FMDV	14	[43,47]
	EV71	34	[43]
Retroviridae **	HIV-1	25–130.8	[43,44,45]

MeV: Measles virus; CDV: Canine distemper virus; PPRV: Peste-des-petits-ruminants virus; MuV: Mumps virus; NDV: Newcastle disease virus; HPIV: Human parainfluenza viruses; NiV: Nipah virus; HMPV: Human metapneumovirus; HRSV: Human respiratory syncytial virus; IFV: Infectious flacherie virus; FMDV: Foot-and-mouth disease virus; EV71: Enterovirus 71; HIV-1: Human immunodeficiency virus type 1.
